# Association between Fetal Adipokines and Child Behavioral Problems at Preschool Age: The Hokkaido Study on Environment and Children’s Health

**DOI:** 10.3390/ijerph15010120

**Published:** 2018-01-11

**Authors:** Machiko Minatoya, Sachiko Itoh, Atsuko Araki, Naomi Tamura, Keiko Yamazaki, Chihiro Miyashita, Reiko Kishi

**Affiliations:** Hokkaido University Center for Environmental and Health Sciences, Kita 12, Nishi 7, Kita-ku, Sapporo 060-0812, Japan; mminatoya@cehs.hokudai.ac.jp (M.M.); vzbghjn@den.hokudai.ac.jp (S.I.); aaraki@cehs.hokudai.ac.jp (A.A.); ntamura@cehs.hokudai.ac.jp (N.T.); kyamazaki@cehs.hokudai.ac.jp (K.Y.); miyasita@med.hokudai.ac.jp (C.M.)

**Keywords:** Strengths and Difficulties Questionnaire (SDQ), adipokines, child behavioral problems, birth cohort study

## Abstract

Studies have suggested associations between maternal obesity and mental health problems of their children. However, the underlying mechanism is largely unknown. A possible mechanism can be via inflammatory states and the other possible mechanism is metabolic hormone-induced programming. Cross-talk between adipokines, including inflammatory cytokines and metabolic hormones secreted from adipose tissue and the central nervous system needs to be further investigated to elucidate the mechanism. Thus, the aim of this study was to investigate the association between fetal adipokine levels and child behavioral problems at preschool age. Cord blood adiponectin, leptin, tumor necrosis factor-α (TNF-α) and interleukin 6 (IL-6) levels were measured and child behavioral problems were assessed using the Strengths and Difficulties Questionnaire at preschool age. Logistic regression models adjusted by related maternal factors were performed to examine the association between cord blood adipokines and child behavioral problems. Three hundred and sixty-one children were included in the final analysis. A significant association between decreased hyperactivity/inattention and increased leptin was found (OR = 0.22, 95% CI: 0.06–0.89). Cord blood adiponectin, TNF-α and IL-6 levels were not associated with child behavioral problems. Our findings suggested that cord blood adipokines, particularly, leptin level, may be a predictor of hyperactivity/inattention problems at preschool age.

## 1. Introduction

Many studies have examined the associations between maternal obesity and a wide range of mental health problems of their children, including emotional and behavioral problems [1,2,3,4]. In general, these studies support an association between maternal BMI and poor cognitive performance and increased risk of developing depression and anxiety, however, the relationship between maternal obesity and the risk of developing autism spectrum disorder (ASD) and attention deficit and hyperactivity disorder (ADHD) in their children is currently less clear [1]. We previously reported that maternal pre-pregnancy BMI ≥ 30 kg/m^2^ was a risk factor for increased child behavioral problems [5]. However, a mechanism connecting observed child behavioral problems and maternal obesity is largely unclear at this time. 

One of the possible mechanisms to explain this can be inflammatory status. Obesity is considered a state of chronic inflammation and secretion of inflammatory biomarkers, including interleuikin-6 (IL-6) and tumor necrosis factor-α (TNF-α) [6]. It has been suggested that elevated levels of inflammatory markers were associated with ASD, ADHD and other neurodevelopmental disorders [3]. Thus, inflammation is an important potential mechanism. Metabolic hormone-induced programming can be another possible mechanism. It is known that there is maternal fetal transmission of leptin, a metabolism-related hormone [7]. Several studies have suggested that leptin levels are associated with psychopathology [8]. Lappas et al. found that leptin activated proinflammatory cytokine release and phospholipid metabolism in human placenta and suggested that leptin may indirectly influence brain development [9].

There have been studies that investigated levels of metabolic hormones, including adiponectin and leptin, in association with mental and neurobehavioral problems such as ASD [10,11] and ADHD [12,13]. These studies have suggested that changes in lipid metabolism are a key to understanding the etiology of neurobehavioral problems, yet the mechanism is mostly unknown. Cross-talk between adipokines, including inflammatory cytokines and metabolic hormones secreted from adipose tissue and the central nervous system needs to be further investigated. Fetal adipokines may potentially contribute to child neurobehavioral development. Thus, the aim of this study was to investigate a possible association between fetal adipokine levels and child behavioral problems at preschool age in a prospective study.

## 2. Methods

### 2.1. Study Population

**Figure 1:**
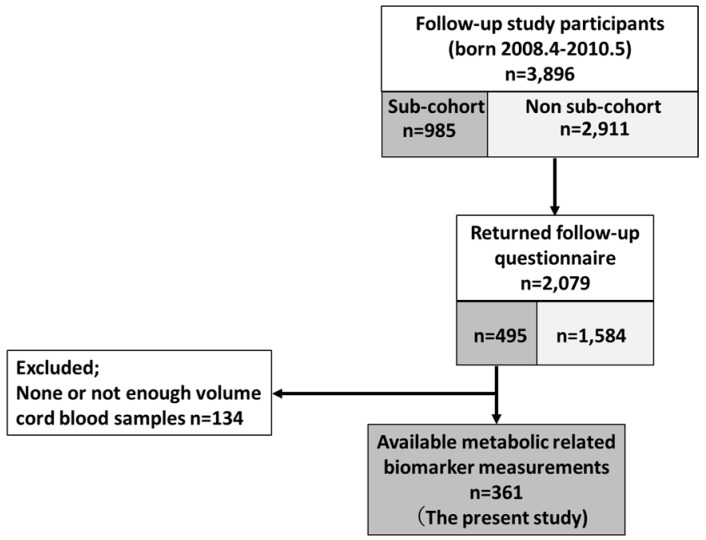
Selection of the study population. The sub-cohort population (23.3% of the whole cohort population) designated for exposure and/or biomarker assessments was randomly selected from the whole cohort population.

This study was one of the follow-up studies of a prospective birth cohort study, the Hokkaido Study on Environment and Children’s Health. The details of the cohort profile can be found elsewhere [14,15,16]. Briefly, the whole cohort included 20,926 pregnant women who enrolled in the study from 2003–2012. A sub-cohort population (23.3% of the whole cohort) designated for exposure and/or biomarker assessments was determined. It is considered to be effective to apply sub-cohort study design for exposure and biomarker assessment. The details of the sub-cohort population can be found elsewhere [17]. The follow-up study targeted those who were born between April 2008 and May 2010 (n = 3896) and have reached the age of 5 years. Strength and Difficulties Questionnaire (SDQ) to assess child neurobehavioral development was distributed via mail to the follow-up study population. Among 3896 participants in the follow-up study, 2079 responded by the end of May 2016. Among the 2079 with valid responses, 495 were from the sub-cohort population. Among the 495 sub-cohort population, we excluded those who did not have cord blood samples for biomarker measurements (n = 134), thus, for the final analysis, 361 participants were included. The flowchart of how the study population was selected was shown in Figure 1. 

This study was conducted with the informed consent of all participants on written forms. The protocol used in this study was approved by the Institutional Ethical Board for epidemiological studies at the Hokkaido University Graduate School of Medicine and Hokkaido University Center for Environmental and Health Sciences on 22 March 2012 (Project ID code; 17-90).

### 2.2. Behavioral Problem Assessment

The Japanese parent-report version of SDQ [18] was distributed via mail to the participants. Parents were asked to fill out the SDQ, which included 25 items on specific strengths and difficulties with an overall rating of whether their child had behavioral problems. Each item has three response categories: (0) not true; (1) somewhat true and (2) certainly true. It includes five subscales (conduct problems, hyperactive/inattention, emotional problems, peer problems and prosocial behavior problems). All subscales excluding pro-social behavior problems were summed to assess the behavioral problems and the total difficulties score (TDS) ranged from 0–40 [19]. Higher scores denote greater problems. SDQ was designed for a broad range of children, age 3–16 years and well-validated tool of childhood mental health [19,20].

We applied score bandings of the Japanese version of SDQ, children with TDS 0–12 were defined as normal, 13–15 were as borderline and 16–40 were as clinical. In this study, children scored ≥13 were defined as likelihood of behavioral problem [18]. For the subscales, the following cut-offs were applied; Conduct problems: 0–3 = normal, 4 = borderline, 5–10 = clinical; Hyperactivity/inattention: 0–5 = normal, 6 = borderline, 7–10 = clinical; Emotional problems; 0–3 = normal, 4 = borderline, 5–10 = clinical; Peer problems: 0–3 = normal, 4 = borderline, 5–10 = clinical; Prosocial behavior; 6–10 = normal, 5 = borderline, 0–4 = clinical [18]. SDQ total and subscale scores were dichotomized comparing the children with borderline and clinical scores with normal children.

### 2.3. Adipokine Measurements

Cord blood samples were obtained at delivery and stored at −80 °C until the analysis. We measured total and high molecular weight (HMW) adiponectin and leptin, TNF-α, and IL-6 levels in cord blood were measured. Both total and HMW adiponectin levels were determined by Enzyme Linked ImmunoSorbent Assay (ELISA) using Human Adiponectin Assay kit from Sekisui Medical Co. Ltd. (Tokyo, Japan). Leptin levels were determined by radioimmunoassay (RIA) using a Human Leptin RIA kit from Linco Research Inc. (St. Charles, MO, USA). TNF-α levels were determined by Chemiluminescent Enzyme Immunoassay (CLEIA) using a Quanti Glo Human TNF-α Chemiluminescent Immunoassay 2nd generation kit from R&D Systems (Minneapolis, MN, USA). IL-6 levels were also determined by CLEIA using a Quanti Glo Human IL-6 Immunoassay 2nd generation kit from R&D Systems. All the analyses were conducted at LSI Medience (Tokyo, Japan) according to the operation manual. The limit of detection (LOD)s of adiponectin was 0.39 μg/mL, of leptin was 0.5 ng/mL, of TNF-α was 0.55 pg/mL, and of IL-6 was 0.3 pg/mL. Intra- and inter-assay CVs for total adiponectin were 7.6–9.1% and 7.8–10.1%, for HMW adiponectin were 6.0–9.2% and 6.8–11.6%, for leptin were 2.8–5.3% and 6.3–8.1%, for TNF-α were 3.2–3.6% and 5.3–6.2%, and for IL-6 were 1.5–7.4% and 3.5–10.5%, respectively.

### 2.4. Covariates

Information on covariates were obtained from questionnaires which were filled out by pregnant women at the 1st trimester, birth records and follow-up questionnaires at age of SDQ completed. Covariates were determined based on both the previous literature [5,21,22,23] and statistical significance (*p* < 0.05) by *t*-test or chi square test as follows; parity, maternal smoking at the 1st trimester, maternal age, maternal education, annual family income at SDQ completed and child sex. 

### 2.5. Statistical Analysis

Cord blood adipokine levels did not distribute normally, thus these values were log10 transformed. The values of adipokine below LOD were replaced as one half the value of LOD for statistical analyses. The logistic regression models were adjusted for the above-mentioned covariates. The odds (ORs) were given for one unit increase on log10 scale of adipokine levels. Statistical analyses were performed using SPSS 22.0J (IBM Japan, Tokyo, Japan). Two side *p* value < 0.05 was considered statistically significant.

## 3. Results

A comparison of the characteristics of participants in the normal and borderline/clinical groups is shown in Table 1. All the characteristics shown in the Table 1, except maternal age and child sex, were not significantly different between the normal and borderline/clinical groups. The mean maternal age was younger in the borderline/clinical group (30.7 ± 4.6) compared to the normal group (32.1 ± 4.3). The borderline/clinical group included higher percentage of boys (63.9%) compared to the percentage in the normal group (49.7%). 

The prevalence of obese (BMI ≥ 25 kg/m^2^) was 8% overall, and no difference was found between the normal and borderline/clinical groups. A comparison of characteristics between follow-up study population and the present study was shown in Table S1 (Supplementary Materials). The distribution of adipokine levels in cord blood is shown in Table 2. 

The median levels of total and HMW adiponectin and leptin were significantly higher in girls compared to these of boys (Table S2). The median level of IL-6 was significantly higher in boys (Table S2). The number of children in borderline/clinical groups of total and each subscale was shown in Table 3. The percentage of children in the borderline/clinical range of TDS was 16.9%. Boys showed a higher prevalence of having problems in TDS, hyperactivity/inattention, and prosocial behavior problems compared to girls. The association between cord blood adipokine levels and child behavioral problems are shown in Table 4. After adjustments, association between decreased hyperactivity/inattention and increased leptin was still significant (OR = 0.23, 95% CI: 0.06–0.89). Increased leptin was marginally associated with prosocial behavioral problems (OR = 0.38, 95% CI: 0.12–1.17) without statistical significance. Cord blood TNF-α and IL-6 levels were not associated with any of the child behavioral problems. The results of stratification by child sex are shown in Table S3. Increased leptin was significantly associated with decreased total problems and hyperactivity/inattention in boys. No significant association was found in girls. 

## 4. Discussion

We found that increased cord blood leptin level was associated with decreased hyperactivity/inattention at preschool age. To our knowledge, this is the first prospective epidemiological study investigated associations between fetal adipokine levels and child behavioral problems. Child behavioral problems in association with obesity, overweight and high BMI have been reported through cross-sectional studies [24,25,26]. Similarly, several previous studies investigated child behavioral problems and cognitive and motor development in association with leptin levels in cross-sectional studies [27,28]. However, these cross-sectional studies could only provide relationships between obesity or biomarker levels and child mental and behavioral problems at one point, but they cannot find causal relationships.

The mean cord blood adiponectin levels of this study (17.1 µg/mL) was in a similar range as previous studies (21.3 µg/mL for a Taiwanese study, and 18.23 µg/mL for a Canadian study, respectively) [29,30]. Contrary, the mean leptin level of this study (4.9 ng/mL) was comparable to levels in a Taiwanese study (4.6 ng/mL) [30], however, much lower compared to the study in Canada (19.8 ng/mL) [29]. Adiponectin and leptin levels vary among ethnicities according to the literature [31], this could explain relatively the lower leptin among the Asian population. Additionally, it is possible that difference in the prevalence of overweight women in this study and others may explain varied leptin levels among studies. The median level of cord blood TNF-α in this study (2.45 pg/mL was lower compared to the previous report from Hong Kong (7.44 pg/mL) [32] and from Taiwan (5.47 pg/mL) [33]. The median level of cord blood IL-6 in this study (1.06 pg/mL) was slightly higher but comparable to the report from Hong Kong (0.65 pg/mL) [32], however, lower than levels in Taiwanese (3.70 pg/mL) [33].

The results from this study suggested that fetal leptin levels may possibly be a predictor of child hyperactivity/inattention problems at preschool age even after controlling for maternal pre-pregnancy BMI. In this study, increased maternal BMI was correlated with increased cord blood leptin levels (Table S2) and increased leptin levels were associated with reduced risk of child hyperactivity/inattention problems. Two of the previous cross-sectional studies found relationships between higher leptin levels and more problems or decrease in cognitive development in children, which was different from our finding. One possible reason for the difference was the difference in the study design. Other possible reason was the difference in the prevalence of overweight. According to the Global Health Observatory data of 2015 from the World Health Organization (WHO), both countries in previous studies (Brazil and Germany) have much higher prevalence of overweight among females (74% and 75%, respectively), whereas prevalence of overweight was only 20% in Japan. Moreover, the trend of prevalence of overweight differed between these two countries and Japan. The prevalence increased in these two countries, contrary, it decreased in Japan. This difference in prevalence of overweight could contribute to different findings among studies. Leptin is known to play a critical role in gestation. The placenta synthesizes leptin, as indicated by the presence of high amount of leptin messenger RNA [34,35]. Other possible sources of leptin for the fetus include fetal membranes and the umbilical cord that co-express leptin and leptin receptor genes during pregnancy [36] and amnion cells that secret leptin into the circulating amniotic fluid [37]. Newborn infant leptin levels are much higher than that of children and adults [38] and the levels decreases significantly after birth [39]. Elevated cord blood leptin levels are associated with increased adiposity and hyperleptinemia of offspring [40].

Leptin levels were not only associated with physiological changes, but may also affect the brain development of the fetus [8]. Although studies have suggested the importance of fetal leptin levels regarding fetal development, little is known in human studies and thus, animal studies are necessary. Leptin has been well studied its role of regulating neuroendocrine system and brain development [41]. In human studies, leptin treatment increased grey matter concentration in areas such as the anterior cingulate gyrus, inferior parietal lobule, and the cerebellum which have roles in emotion, attention and motivation [42] and improved cognitive development [43]. The amygdala is well known for its role in anxiety and stress response and the presence of leptin receptors, and their projections to this brain region indicate that leptin may play a role in the mediation of emotions and behaviors [8]. Leptin regulates several neurotransmitter systems and brain regions critical in behavioral regulation in both rodents and human [44,45]. The emerging picture of leptin interaction with lateral hypothalamic area (LHA) suggests that the LHA is not merely regulating feeding, but is a crucial integrator of energy balance and motivated behavior [46]. It is possible that leptin has effects on fetal development and psychopathology via inflammation according to some evidences [9,47]. However, in this study, cord blood inflammatory biomarker levels including TNF-α and IL-6 were found to be not associated with child behavioral problems. This indicated that cord blood leptin level was independently associated with child behavioral problems.

In animal models [48], neonatal leptin deficiency increased cerebral cortex leptin receptor expression and reduces frontal cortex volumes in association with increased adult locomotor activity. Similarly, falling levels of leptin resulted in increased activity levels in rat model [49]. Furthermore, in growth restricted male mice, physiologic leptin replacement improves adult neuro outcomes [50]. Our results agree with these findings from animal studies.

In an adult study, higher plasma adiponectin levels were associated with neurodegeneration and cognitive decline [51]. Contrary, a recent review article mentioned that adiponectin had several protective functions in the peripheral tissues including insulin sensitizing, anti-inflammatory and anti-oxidative effects that may benefit neurodegenerative diseases [52]. The role of adiponectin is still controversial. Moreover, no prospective study has shown the association between adiponectin and child neurobehavioral problems to our knowledge.

The previous study showed that elevated levels of IL-6, but not TNF-α were significantly associated with a decrease in motor score in the first year of life [51]. Their finding suggested that markers of inflammation could serve as prognostic indicators. However, in this study, we did not find any association between either IL-6 or TNF-α and child behavioral problems. Even though both cross-sectional and prospective studies have been conducted, the findings were inconclusive. Thus, more longitudinal studies are needed to confirm whether these biomarkers can be a predictor of child neurobehavioral problems or not.

The strength of this study was a prospective study design and it was able to report longitudinal associations. Our study also has limitations. Although this study was population based, the findings may not be fully representative for the total population with regard to sociodemographic characteristics. The percentages of parental education ≥13 years were higher in this study population compared to the follow-up study population (n = 3896), however, not very different from the returned SDQ population (n = 2079). The percentage of annual family income during pregnancy ≥5 million Japanese yen was also higher in this study population compared to the follow-up population but not that much from the returned SDQ population. This implied that null bias existed between the population with (the present study, n = 361) and without (returned SDQ, n = 2079) adipokine measurements. Contrary, the percentage of maternal smoking during pregnancy was much lower in this study population compared to the follow-up study population and also slightly lower compared to the returned SDQ population (Table S1). Therefore, the present study population may represent a higher socioeconomic status population. Since parental education and annual family income were inversely correlated with SDQ scores and maternal smoking during pregnancy was positively associated with SDQ scores (Table S4), we may have underestimated the effects. One other issue was that we did not assess maternal mental health including anxiety or depression in this study. Maternal mood and mental health may contribute to child behavioral problems, however, we were unable to control for the statistical analysis. In addition, maternal history of hyperactivity/inattention was not obtained and not controlled in the statistical analysis. Thus, our findings may possibly be hindered. Finally, we should address that the prevalence of obese pregnant women in this study was not high (8%), and thus, this study population may not be appropriate to examine maternal obesity induced inflammation programing of offspring neurodevelopmental disorders.

## 5. Conclusions

Our findings suggest that cord blood adipokine, particularly, leptin levels can be a predictor of hyperactivity/inattention problem at preschool age. In addition, this was the first study to investigate association between cord adiponectin levels and child behavioral problems in a prospective manner. Further prospective epidemiological studies are warranted to confirm whether these biomarkers can be predictors of child neurobehavioral outcomes.

## Figures and Tables

**Table 1:** Comparison of characteristics of participants in normal and borderline/clinical groups of the total difficulties score (TDS).

Characteristics		All (n = 361)	Normal (n = 300)	Borderline/Clinical (n = 61)	*p*-Value ^a^
		Mean ± SD or n (%)			
**Parent**					
Maternal age (years)		31.9 ± 4.4	32.1 ± 4.3	30.7 ± 4.6	0.022
Maternal pre-pregnancy BMI (kg/m^2^)		20.8 ± 2.7	20.8 ± 2.7	21.1 ± 3.1	0.391
	<18.5	60 (16.6)	47 (15.7)	13 (21.3)	0.439
	18.5–24.99	265 (73.4)	224 (74.7)	41 (67.2)	
	≥25	29 (8.0)	23 (7.7)	6 (9.8)	
Parity	Primipara	143 (39.6)	115 (38.3)	28 (45.9)	0.174
Maternal education (years)	≤12	141 (39.1)	114 (38.0)	27 (44.3)	0.403
	≥13	216 (59.8)	182 (60.7)	34 (11.3)	
Alcohol consumption during pregnancy	Yes	40 (11.1)	35 (11.7)	5 (8.2)	0.448
Smoking during pregnancy	Yes	16 (4.4)	12 (4.0)	4 (6.6)	0.342
Paternal age (years)		32.8 ± 6.4	33.1 ± 6.7	31.6 ± 4.7	0.118
Paternal education (years)	≤12	137 (38.0)	117 (39.0)	20 (32.8)	0.324
	≥13	220 (60.9)	179 (59.7)	41 (67.2)	
Annual family income at SDQ completed (million JPY)	<5	170 (47.1)	137 (45.7)	33 (54.1)	0.154
	≥5	175 (48.5)	151 (50.3)	24 (39.3)	
**Child**					
Sex	Boys	188 (52.1)	149 (49.7)	39 (63.9)	0.042
	Girls	173 (47.9)	151 (50.3)	22 (36.1)	
Birth weight (g)		3038 ± 358	3036 ± 346	3046 ± 402	0.833
Birth length (cm)		48.9 ± 1.9	48.8 ± 1.8	49.2 ± 2.0	0.218
Gestational age (days)		275 ± 8	275 ± 8	275 ± 8	0.528
Age at SDQ answered (months)		66.9 ± 6.1	66.8 ± 6.1	67.3 ± 6.3	0.602

SD: standard deviation, BMI: body mass index, JPY: Japanese yen. ^a^ *t*-test or chi square test.

**Table 2:** Cord blood adipokine levels.

Adipokines	n	Median IQR (25–75th)
Total adiponectin (µg/mL)	361	17.1 (12.8–20.8)
HMW adiponectin (µg/mL)	361	12.8 (8.1–14.9)
Leptin (ng/mL)	357	4.9 (3.1–8.0)
TNF-α (pg/mL)	353	2.45 (1.89–3.19)
IL-6 (pg/mL)	350	1.06 (0.62–2.60)

IQR: inter quartile range, HMW: high-molecular-weight, TNF-a: tumor necrosis factor alpha, IL-6: interleukin 6.

**Table 3:** Number of children in borderline/clinical groups of total and each subscale.

Scales	n (%) in Borderline/Clinical			*p*-Value ^a^
	All	Boys	Girls	
TDS	61 (16.9)	39 (20.7)	22 (12.7)	0.042
Conduct problems	57 (15.8)	36 (19.1)	21 (12.1)	0.068
Hyperactivity/inattention	47 (13.0)	34 (18.1)	13 (7.5)	0.003
Emotional symptoms	58 (16.1)	29 (15.4)	29 (16.8)	0.730
Peer problems	25 (6.9)	12 (6.4)	13 (7.5)	0.672
Prosocial behavior problems	70 (19.4)	47 (25.0)	23 (13.3)	0.005

^a^ chi square test.

**Table 4:** Association between cord blood adipokine levels and child behavioral problems.

Adipokines	OR (95% CI)					
	TDS	Conduct Problems	Hyperactivity/Inattention	Emotional Symptoms	Peer Problems	Prosocial Behavior Problems
Total adiponectin	0.97 (0.12–7.77)	0.20 (0.02–1.73)	1.11 (0.12–10.26)	0.46 (0.06–3.75)	2.13 (0.11–41.93)	0.23 (0.03–1.68)
HMW adiponectin	0.87 (0.19–4.05)	0.37 (0.08–1.78)	0.70 (0.14–3.55)	0.49 (0.10–2.30)	1.68 (0.19–14.77)	0.44 (0.10–1.91)
Leptin	0.44 (0.14–1.44)	0.99 (0.30–3.24)	0.22 (0.06–0.89) *	0.84 (0.27–2.62)	0.63 (0.14–2.89)	0.40 (0.13–1.25)
TNF-α	1.14 (0.26–5.03)	0.73 (0.15–3.58)	1.63 (0.37–7.09)	0.53 (0.09–3.10)	3.88 (0.83–18.13) ^+^	1.54 (0.41–5.82)
IL-6	0.85 (0.49–1.46)	1.21 (0.77–1.90)	0.91 (0.52–1.61)	0.99 (0.60–1.64)	1.36 (0.77–2.39)	1.30 (0.85–1.97)

Levels of metabolic related biomarker were log_10_ transformed. ^+^ *p* < 0.10, * *p* < 0.05. Adjusted with parity, maternal smoking at first trimester, maternal pre-pregnancy BMI, maternal age, annual family income at SDQ completed and child sex.

## Supplementary Materials

The following are available online at www.mdpi.com/1660-4601/15/1/120/s1, Table S1. Comparison of characteristics among the follow-up study population (n = 3896), returned SDQ (n = 2079) and the present study (n = 361); Table S2. Association between participants’ characteristics and adipokine levels in cord blood; Table S3. Association between cord blood adipokine levels and child behavioral problems stratified by child sex; Table S4. Spearman’s correlation co efficient between characteristics of the present study population and total difficulties scores of SDQ (n = 2079).
