# Supplementary material for: Association between Fetal Adipokines and Child Behavioral Problems at Preschool Age: The Hokkaido Study on Environment and Children’s Health
Source: Int J Environ Res Public Health. 2018 Jan 11;15(1):120. doi: 10.3390/ijerph15010120 (PMC5800219; doi:10.3390/ijerph15010120)
Supplement: Supplementary file 1 [file ijerph-15-00120-s001.pdf]

Association Between Fetal Adipokines and Child Behavioral Problems at Preschool Age: The Hokkaido Study on Environment and Children's Health

Machiko Minatoya [1], Sachiko Itoh [1], Atsuko Araki [1], Naomi Tamura [1], Keiko Yamazaki [1], Chihiro Miyashita [1] and Reiko Kishi [1],*

[1] Hokkaido University Center for Environmental and Health Sciences, Kita 12, Nishi 7, Kita-ku, Sapporo 060-0812, Japan; mminatoya@cehs.hokudai.ac.jp (M.M.), vzbghjn@den.hokudai.ac.jp (S.I.), aaraki@cehs.hokudai.ac.jp (A.A.), ntamura@cehs.hokudai.ac.jp (N.T.), kyamazaki@cehs.hokudai.ac.jp (K.Y.), miyasita@med.hokudai.ac.jp (C.M.)

* Correspondence: rkishi@med.hokudai.ac.jp; Tel.: +81-11-706-4746

Received: 12 October 2017; Accepted: 09 January 2018; Published: date

Table S1. Comparison of characteristics among the follow-up study population (n = 3896), returned SDQ (n = 2079) and the present study (n = 361).

Characteristics		Mean ± SD or n (%)		
		**Follow-up Study (n = 3896)**	**Returned SDQ (n = 2079)**	**The Present Study (n = 361)**
**Parent**				
Maternal age (years)		30.7 ± 4.9	31.4 ± 4.6	31.9 ± 4.4
Maternal pre-pregnancy BMI (kg/m^2)		21.2 ± 3.4	21.1 ± 3.3	20.8 ± 2.7
Parity	Primipara	1548 (40.1)	854 (41.1)	143 (39.6)
Maternal education (years)	≤12	1780 (46.1)	822 (39.5)	141 (39.1)
	≥13	1943 (50.3)	1208 (58.1)	216 (59.8)
Alcohol consumption during pregnancy	Yes	404 (10.5)	220 (10.6)	40 (11.1)
Smoking during pregnancy	Yes	376 (9.7)	136 (6.5)	16 (4.4)
Paternal age (years)		32.5 ± 5.7	33.0 ± 5.5	32.8 ± 6.4
Paternal education (years)	≤12	1768 (45.8)	880 (42.3)	137 (38.0)
	≥13	1907 (49.4)	1138 (54.7)	220 (60.9)
Annual Family income during pregnancy (million JPY)	<5	2160 (56.0)	1137 (54.7)	185 (51.2)
	≥5	1099 (28.5)	671 (32.3)	131 (36.3)
	Missing	601 (15.6)	271 (13.0)	45 (12.5)
**Child**				
Sex	Boys	1929 (49.5)	1032 (49.6)	188 (52.1)
Birth weight (g)		2997 ± 451	3005 ± 422	3038 ± 358
Birth length (cm)		48.7 ± 4.5	48.7 ± 2.2	48.9 ± 1.9
Gestational age (days)		273 ± 12	274 ± 11	275 ± 8

SDQ: Strengths and Difficulties Questionnaire; JPY: Japanese yen; BMI: body mass index; SD: standard deviation.

Table S2. Association between participants' characteristics and adipokine levels in cord blood.

Characteristics		Total Adiponectin (µg/ml)	HMW Adiponectin (µg/ml)	Leptin (pg/ml)	TNF-α (pg/ml)	IL-6 (pg/ml)
Maternal age (years)		0.043	0.065	-0.075	-0.063	0.028
Maternal pre-pregnancy BMI (kg/m^2)		0.113 *	0.062	0.146 **	-0.057	0.047
Parity	Primipara	16.9 (12.0–21.0)	11.3 (7.5–14.9)	5.6 (3.2–8.8)	2.46 (1.88–3.30)	1.33 (0.79–3.36)
	Multipara	17.6 (13.3–21.2)	11.3 (8.6–15.0)	4.5 (2.9–7.4)	2.49 (1.95–3.18)	0.91 (0.56–1.88)
Maternal education (years)	≤12	17.3 (13.9–21.2)	11.3 (8.7–14.8)	5.1 (3.2–8.0)	2.51 (1.93–3.17)	1.00 (0.57–2.26)
	≥13	16.9 (12.3–20.7)	11.3 (8.0–15.0)	4.9 (3.1–8.1)	2.42 (1.85–3.29)	1.12 (0.66–3.22)
Maternal alcohol consumption during pregnancy	Yes	17.7 (11.5–21.2)	11.4 (7.6–14.6)	5.3 (3.2–7.5)	2.36 (1.83–3.18)	0.87 (0.52–2.06)
	No	17.1 (12.8–20.8)	11.0 (8.2–15.0)	4.8 (3.1–8.2)	2.46 (1.89–3.19)	1.09 (0.63–2.73)
Maternal smoking during pregnancy	Yes	16.3 (9.7–20.8)	11.1 (6.2–14.5)	4.2 (2.2–5.7)	2.69 (2.04–3.40)	1.04 (0.72–1.84)
	No	17.6 (12.7–21.4)	11.4 (8.1–15.3)	5.4 (3.2–8.3)	2.46 (1.89–3.19)	1.11 (0.63–2.79)
Annual family income during pregnancy (million JPY)	<5	16.7 (12.3–21.2)	11.0 (7.8–14.9)	4.8 (3.0–8.2)	2.44 (1.90–3.19)	1.06 (0.63–2.78)
	≥5	17.8 (13.5–20.5)	11.4 (8.6–15.3)	4.7 (3.1–7.6)	2.50 (1.88–3.19)	1.03 (0.60–2.23)
Child sex	Boys	16.5 (11.9–20.0)	10.1 (7.4–14.3)	4.4 (2.7–7.2)	2.55 (1.96–3.36)	1.11 (0.67–3.78)
	Girls	18.1 (13.1–21.4)	11.9 (9.0–15.7)	5.5 (3.3–9.3)	2.35 (1.84–3.03)	1.02 (0.60–1.92)
Birth weight (g)		0.235 **	0.215 *	0.325 *	-0.097	0.036
Birth length (cm)		0.128 *	0.123 *	0.200 **	-0.100	0.004
Gestational age (days)		0.095	0.097	0.243 **	-0.176 **	0.179 **

TNF-α: tumor necrosis factor-α; HMW: high molecular weight; BMI: body mass index; JPY: Japanese yen. Spearman's correlation coefficient or median (IQR). ** $p < 0.001$, * $p < 0.05$.

Table S3. Association between cord blood adipokine levels and child behavioral problems stratified by child sex.

Adipokines	OR (95% CI)					
	**TDS**	**Conduct Problems**	**Hyperactivity/Inattention**	**Emotional Symptoms**	**Peer Problems**	**Prosocial Behavior Problems**
Boys						
Total adiponectin	0.64 (0.05, 7.57)	0.20 (0.02, 2.33)	0.43 (0.04, 4.96)	2.09 (0.10, 45.60)	N/A	0.20 (0.02, 2.11)
HMW adiponectin	0.47 (0.07, 2.93)	0.26 (0.04, 1.66)	0.34 (0.05, 2.09)	1.62 (0.17, 15.74)	N/A	0.39 (0.07, 2.27)
Leptin	0.21 (0.04, 0.99) *	1.16 (0.25, 5.40)	0.09 (0.02, 0.48) *	1.12 (0.21, 6.03)	N/A	0.39 (0.09, 1.64)
TNF-α	0.84 (0.15, 4.81)	0.11 (0.01, 1.14) $^{+}$	2.16 (0.47, 10.00)	0.93 (0.15, 5.98)	N/A	2.39 (0.55, 10.28)
IL-6	0.82 (0.44, 1.55)	1.03 (0.59, 1.80)	0.91 (0.49, 1.68)	0.87 (0.43, 1.73)	N/A	1.41 (0.88, 2.26)
Girls						
Total adiponectin	3.09 (0.06, 162.26)	0.27 (0.00, 20.51)	N/A	0.09 (0.00, 2.70)	N/A	0.34 (0.01, 17.02)
HMW adiponectin	4.35 (0.21, 88.61)	1.01 (0.04, 23.75)	N/A	0.14 (0.01, 1.67)	N/A	0.58 (0.04, 9.65)
Leptin	1.21 (0.20, 7.46)	0.78 (0.10, 6.16)	N/A	0.67 (0.13, 3.55)	N/A	0.29 (0.04, 2.14)
TNF-α	3.62 (0.14, 90.80)	N/A	N/A	0.10 (0.00, 4.02)	N/A	0.11 (0.00, 5.38)
IL-6	0.87 (0.30, 2.54)	2.01 (0.79, 5.12)	N/A	1.12 (0.49, 2.54)	N/A	0.93 (0.31, 2.78)

Levels of metabolic related biomarker were $\log_{10}$ transformed. $^{+}$ $p < 0.10$, * $p < 0.05$. Adjusted with parity, maternal smoking at first trimester, maternal pre-pregnancy BMI, maternal age, and annual family income at SDQ completed. N/A; not available for statistical analysis due to small number of cases.

Table S4. Spearman's correlation co efficients between characteristics of the present study population and total difficulties scores of SDQ (n = 2079).

Characteristics	Spearman's rho
Parents	
Maternal age	-0.087 **
Maternal pre-pregnancy BMI	0.068 *
Parity	-0.102 **
Maternal education	-0.103 **
Alcohol consumption during pregnancy	0.037
Smoking during pregnancy	0.108 **
Paternal age	-0.078 *
Paternal education	-0.046 *
Annual family income during pregnancy	-0.103 **
Annual family income at SDQ completed	-0.122 *
Child	
Sex	-0.135 **
Birth weight	0.023
Birth length	0.020
Gestational age	-0.019

* $p < 0.05$, ** $p < 0.01$. SDQ: Strengths and Difficulties Questionnaire; BMI: body mass index.
